# Connectivity and the search for specializations in the language-capable brain

**DOI:** 10.1016/j.cobeha.2017.11.001

**Published:** 2018-06

**Authors:** Rogier B Mars, Nicole Eichert, Saad Jbabdi, Lennart Verhagen, Matthew FS Rushworth

**Affiliations:** 1 Wellcome Centre for Integrative Neuroimaging, Centre for Functional MRI of the Brain (FMRIB), Nuffield Department of Clinical Neurosciences, University of Oxford, Oxford, United Kingdom; 2 Donders Institute for Brain, Cognition and Behaviour, Radboud University Nijmegen, Nijmegen, The Netherlands; 3 Wellcome Centre for Integrative Neuroimaging, Department of Experimental Psychology, University of Oxford, Oxford, United Kingdom

## Abstract

The search for the anatomical basis of language has traditionally been a search for specializations. More recently such research has focused both on aspects of brain organization that are unique to humans and aspects shared with other primates. This work has mostly concentrated on the architecture of connections between brain areas. However, as specializations can take many guises, comparison of anatomical organization across species is often complicated. We demonstrate how viewing different types of specializations within a common framework allows one to better appreciate both shared and unique aspects of brain organization. We illustrate this point by discussing recent insights into the anatomy of the dorsal language pathway to the frontal cortex and areas for laryngeal control in the motor cortex.

## Searching for specializations in the evolution of language

The search for the origin of language has traditionally been interpreted as a search for specializations. Language is the one behavior that is still generally acknowledged to have some uniquely human component and much of the study of language evolution has been a search for the crucial modification that allowed this to appear. However, the study of language is increasingly informed by studies in other species; the mechanisms underlying component aspects of language in related species are informative about our own abilities [1,2^•^].

For this reason, Hauser and colleagues proposed a distinction between the faculty of language in the broad sense (FLB) and the faculty of language in the narrow sense (FLN) [3]. The study of FLB is the study of all aspects of language, including those that are partly present in non-human animals. In contrast, the study of the FLN is concerned with identifying that aspect of language that is truly unique to the human. In this model, it is essential to realize that to understand language one needs to understand both FLB and FLN. Indeed, it might be that the FLN is empty and all language behavior can be explained through a combination of behaviors present in some other species [1]. Language behavior, as such, is not a unitary construct but a combination of abilities that need to be explained and it is the result of these that we call ‘language’.

These realizations apply equally to the study of the anatomical basis of language. Although language is uniquely human, many of its component processes (FLB) rely on circuitry that we share with other primates. Rather than searching for an anatomical silver bullet, some recent models acknowledge that most major pathways of the neocortex subserve processes that are relevant for human language but are not intrinsically specific to it [4,5]. This is similar to proposals recently made for social cognition, in which social behavior is seen as the result of a multitude of systems, perhaps none of which is exclusively devoted to this task [6].

All primate brains share a common blueprint. Specializations occur through modifications in certain lineages or specific species. Analogous to the FLB/FLN argument, a full understanding of the evolution of the neural basis of language requires an understanding of the shared aspects of this blueprint and modifications in the anthropoid, ape, and ultimately human branches of the evolutionary tree. In the domain of language, this work has mostly focused on the neocortex, although the role of subcortical areas in language is increasingly recognized [7].

## Identifying specializations

**Figure 1:**
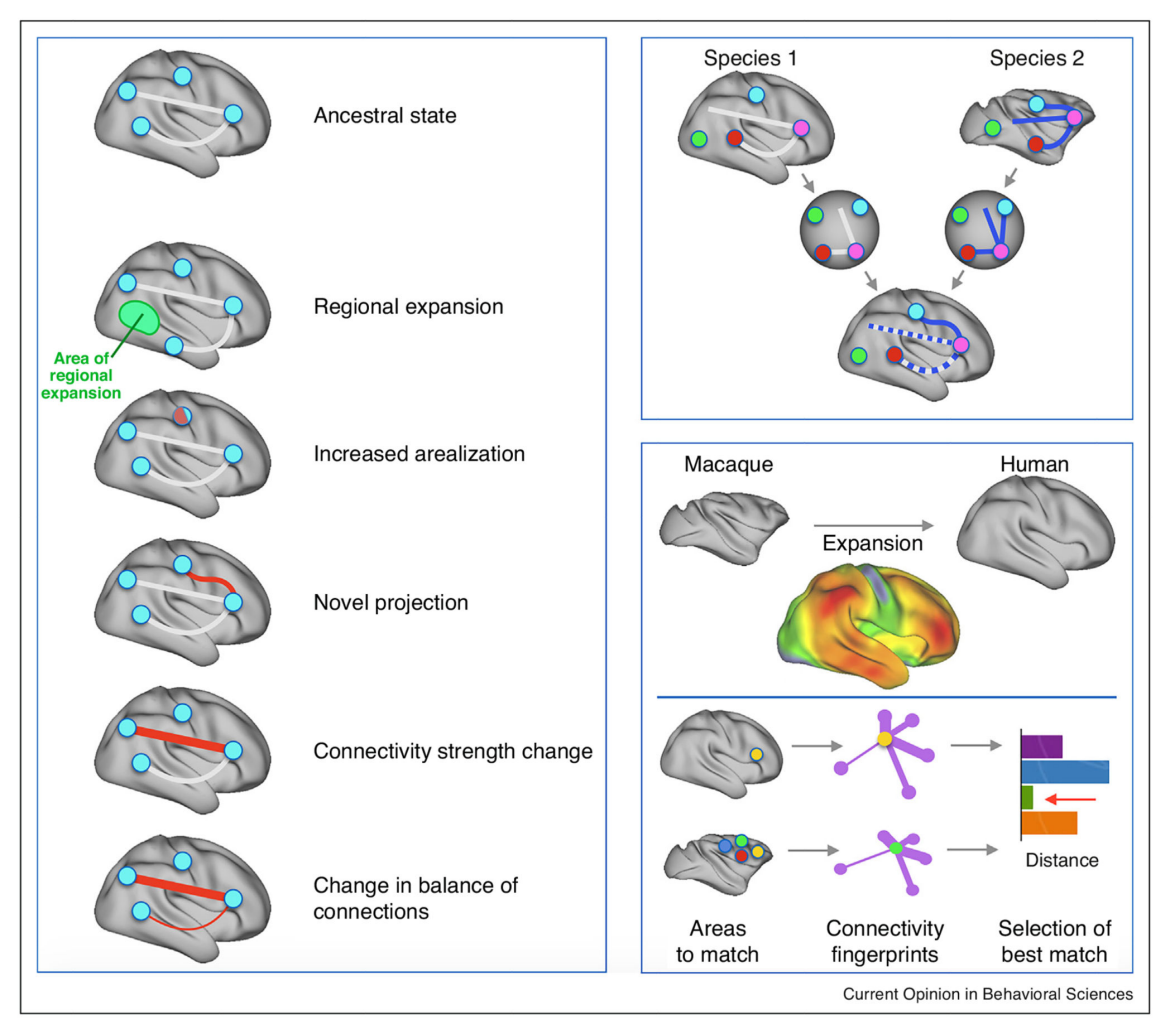
Anatomical specializations. (*left panel*) Anatomical modifications can take many forms, as has been demonstrated in studies of the sensorimotor system and the association cortex [8,9]. Apart from a general increase in the size of the cortical sheet, local expansion might have led to changes in the relation of certain structures; the number of areas might increase; changes in connections can take the form of completely novel projections or a change in the strength of certain connections, potentially leading to a change in the balance of input or outputs of a region. (*top right panel*) To fully understand the differences between two brains it is helpful to place them into a common space. In this schematic example homologous brain regions are overlaid, leading to an assessment of changes in the connections. (*lower right panel*) Two examples of such an approach are surface-based registration based on sulcal anatomy, allowing one to judge local expansions [12], and connectivity fingerprint matching [14^•^], allowing one to compare regions based on their profile of connections with homologous areas.

Cortical modifications can come in many guises [8,9] (Figure 1, left). The most obvious difference across different primate species is the size of the cortical sheet, even when taking into account relative body size. The expansion of the cortical sheet is, however, not uniform. For instance, the human association cortex has preferentially expanded even in comparison with that of great apes [10]. Even in the absence of any other differences, such local expansions can distort the relative locations of other anatomical landmarks.

Apart from differences in the size of the cortical sheet or in the relative size of particular cortical fields, one can look for differences in the number of cortical fields or the connections of homologous fields. Differences in connectivity have been a prominent component of most recent models of language evolution, made possible by the widespread availability of tools to study connections *in vivo* in the human brain [11]. Differences in connections can mean either differences in the strength of existing connections, sometimes changing the balance of inputs from distant regions, or wholly new projections. Functionally, existing anatomical structures can be recruited into new functions due to changes in other parts of the cortex. Only by investigating all these possible differences simultaneously can one build up a complete understanding of the specializations of any given brain.

When comparing various brains it is difficult to assess which types of differences have occurred. Therefore, it can be helpful to project the different samples to a common reference space (Figure 1, right), equating certain features of brain organization to assess how other features then compare across samples. An early example of this approach was provided by studies using surfacebased registration to align brains of different primates based on explicit hypotheses of sulcal homology [12,13]. Equating these sulci provided landmarks that could be overlaid, after which the relative sizes of parts of the cortex could be compared across species. These studies showed that association cortex tends to expand when comparing marmosets to capuchins and macaques and when comparing macaques to humans. There are local hotspots of expansion in temporoparietal, ventrolateral, and medial frontal cortex.

An alternative approach involves so-called ‘connectivity fingerprint matching’ [14^•^]. This approach solves the registration problem by comparing the profile of connections of different areas with known homologs across species. In effect, the approach brings the areas under investigation into a common connectivity space. It has been used to examine the existence of homologies between humans and macaques in frontal [15,16] and temporoparietal cortex [17].

Ultimately, registration between species should be conducted on the basis of multiple features, such as sulcal anatomy, connectivity, functional similarity, and histological properties [18]. Different types of anatomical data are increasingly becoming available for such approaches, either by using different neuroimaging techniques [19,20] or by registering histological atlases to MRI templates [21]. This approach allows one to directly compare different aspects of brain organization and specify the interaction between the different types of specializations. In what follows, we apply this line of inquiry to two of the most studied aspects of language anatomy: the architecture of the longitudinal pathways to the frontal lobe and the laryngeal motor cortex.

## Longitudinal pathways to the frontal lobe

The frontal lobe is connected with other parts of the neocortex via, among others, a series of longitudinal association fibers. So-called dorsal pathways consist of different branches of the superior longitudinal fascicle reaching parietal cortex, and the arcuate fascicle (AF) reaching the inferior parietal and superior temporal cortex. The ventral pathways consist of the uncinate fascicle, connecting anterior temporal and ventrolateral and orbital frontal cortex, and fibers running more dorsally through the extreme capsule.

One of the most influential findings in the comparative anatomy of language concerns the arcuate fascicle. The AF was traditionally seen as the main dorsal white matter pathway between Broca’s and Wernicke’s areas; damage to this pathway was the classical interpretation of conduction aphasia [22]. The advent of diffusion MRI tractography made it possible to study this tract in the living human brain. Following early diffusion MRI tractography reports that human AF differed from what was known from tracer studies of AF in monkeys, Rilling and colleagues [23] used tractography to compare the course of AF in humans, chimpanzees, and macaques. They reported that human AF extends further into the temporal lobe in humans than in other primates. This finding has been very influential, guiding both computational [24] and clinical [25] accounts of language.

**Figure 2:**
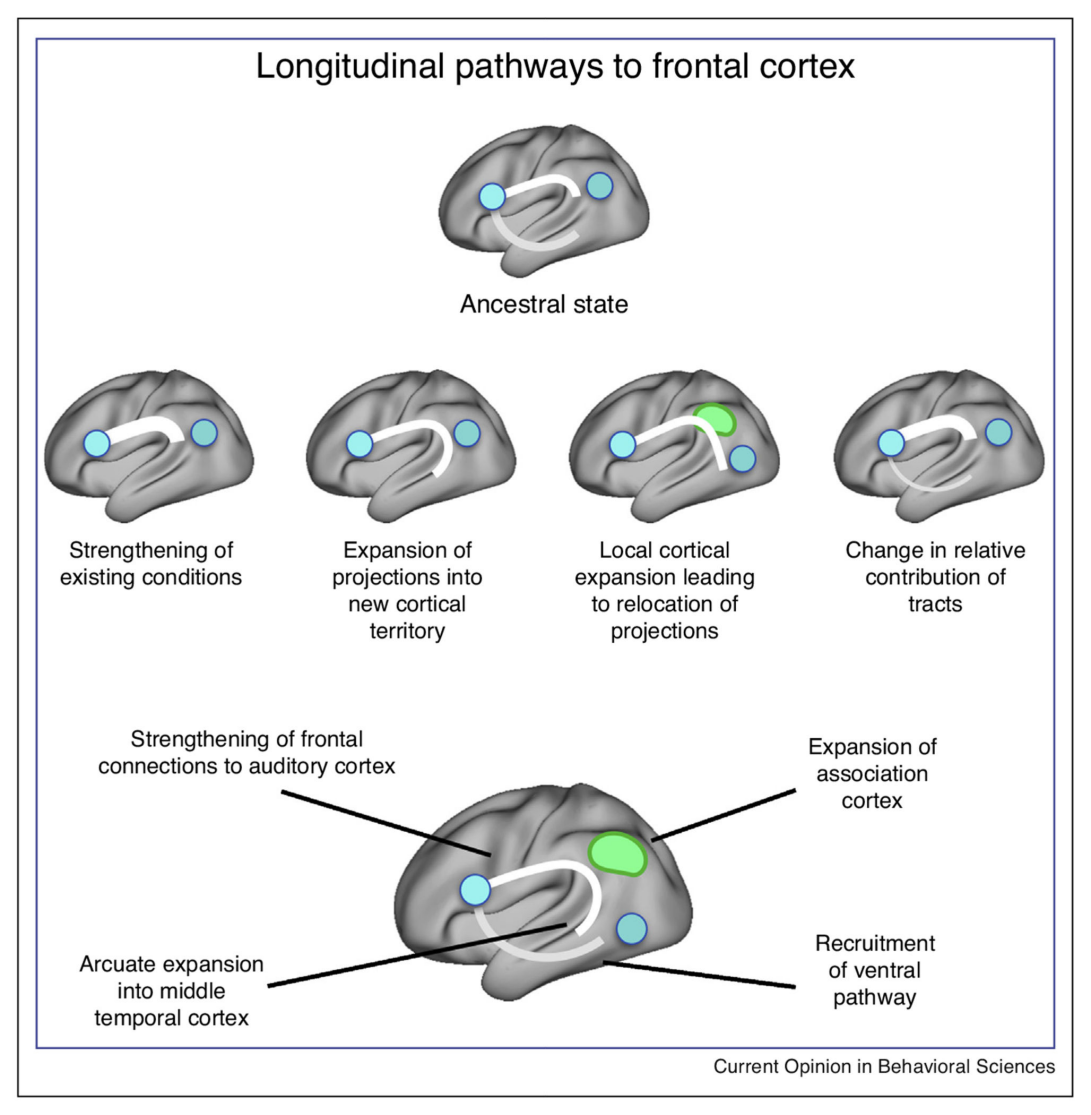
Potential human specializations in longitudinal pathways to frontal cortex. Compared to a hypothesized common ancestor of humans and monkeys that has both ventral (i.e. uncinate and extreme capsule) and weak doral (i.e. arcuate fascicle (AF)) pathways to the frontal cortex (*top*), a number of potential specializations have been proposed to have occurred in the human lineage. These include (*middle*) a change in the strength of the AF and an expansion of projections of the AF into the temporal lobe [23]. However, such changes should be seen in the context of local expansions of the temporoparietal association cortex [12] and recruitment and modification of other existing pathways [30]. All these considerations together lead to a more complete picture of the longitudinal pathways (*bottom*), including both human specializations and features shared with other species.

However, it has proven difficult to define the exact specializations of the human AF (Figure 2). On the one hand, some studies have found evidence that human AF reaches areas beyond the language system, such as dorsal frontal cortex [26], just as it does in macaque tracer studies [27]. On the other hand, recent evidence suggests that macaque AF may interconnect brain regions that had been thought to be specialized targets of human AF such as the superior temporal gyrus and ventrolateral frontal cortex [28^••^], although tractography results suggest such connections are much weaker in macaques than in humans.

The main human specialization of AF is commonly taken to be the extension of posterior projections into the middle and inferior temporal cortex. This result should, however, be seen in the context of the large expansion of temporoparietal cortex [12] and the subsequent reorganization of this part of the cortex [17]. For example, area MT is located much more ventrally in the human temporal cortex than in the macaque due to expansion of the temporoparietal association cortex. This has been shown by direct comparison of myelin maps of the monkey, ape, and human cortex [29]. Given that this area is near some of the projections of the AF, this could mean that in the human there is an extension in the areas that AF interconnects or it could mean that AF interconnects the same areas but the areas are relocated. This hypothesis can be tested by identifying the warp specifying the cortical expansion between macaques and humans and applying it to a map of projections of macaque AF. If relocation accounts for all human AF projections, this should produce a map of human AF. Current results suggest that this is not the case and human AF indeed has novel temporal projections over and above those predicted by local expansion of cortical territory (N. Eichert et al., unpublished data).

Finally, the predominant focus on the dorsal pathways has been challenged by a number of studies highlighting the language-related contribution of ventral tracts between the temporal and frontal lobe [30–32] and within the temporal lobe [33]. Ventral frontal-temporal connections have been suggested to project much wider in the human than macaque brain [34]. However, comparison of ventral fibers between species has been hindered by the use of different methods across species, with original reports using diffusion MRI tractography in humans and tracers in the macaque. More recent studies using tractography in multiple species show a frontal-temporal pathway more similar to that of the human in macaques ([35], but see [36]) and in marmosets [37]. Although the ventral pathway is somewhat more developed in the human, the dorsal pathway may have seen the most dramatic expansion, in essence changing the balance of inputs to the frontal lobe [38]. However, any theory of the evolution of language has to account for the involvement of the ventral pathways both in language and non-language processes. The ventral pathway, although perhaps more conserved than the dorsal pathway, has undoubtedly been recruited to serve specific language-related functions.

## Pathways for vocal learning and vocalization

The ability to modify the production of acoustic features of sounds by imitation and improvisation is a critical component of spoken language acquisition. The evolution of vocal communication in humans requires both the ability to voluntarily control one’s vocal apparatus and the capacity for vocal learning. Vocal learning is rare in the animal kingdom. Research on avian vocal learners has proposed detailed models of its evolution, including the ‘duplication model’ in which motor learning pathways connecting sensory areas duplicate and form connections to brainstem circuits controlling vocalization. It has been proposed that analogous modifications have occurred in the human lineage [39].

As is the case in birds, most non-human primates produce emotional vocalizations, but flexible vocal production is rare. Most primates are able to exert cognitive control over emotional vocalizations [40^•^]. They can inhibit emotional vocalizations and even learn relationships between stimuli and existing vocalizations, but the extent to which they can modify calls or produce novel utterances is unclear (see Ghazanfar and Hage, in this special issue). Humans do this routinely and this is one of the bases of our spoken language. It has been suggested that this is due to increased control over the laryngeal muscles. In the non-human primate such control is at least partially achieved through a distinct laryngeal representation, termed the laryngeal motor cortex (LMC). In the human, functional imaging studies found representations of the laryngeal musculature in the dorsal part of the orofacial motor cortex [41,42]. Consistent with the suggestion of increased laryngeal control in humans, this area in the human might have more direct connections to the nucleus ambiguus, a laryngeal brainstem nucleus ([43], but see [44]).

One possibility is that in humans there has been a duplication of an ancestral primate area concerned with laryngeal control. Neural stimulation studies have suggested the presence of not one but two laryngeal representations in the human motor cortex [45]. The ventral location seems closer to what would be predicted based on LMC’s position in non-human primates. The dorsal one seems to overlap with the region identified in the functional imaging studies discussed above. However, the duplication model is complicated by a number of factors. The human LMC area identified using neuroimaging is located in cytoarchitectonic area 4, rather than area 6 as in the macaque [41]. The proposed homology of human and macaque LMC is thus based on functional criteria but the cytoarchitecture and connectivity are discrepant. This proposal is arguably problematic because, by definition, function cannot be completely the same in language and non-language-capable brains. Finally, some authors have argued that the connectivity of LMC to subcortical nuclei as currently described is not complete, and that as yet undiscovered pathways are required to explain circumstances leading to LMC activation [46].

The neural circuitry related to vocal learning is more straightforward to study comparatively. It would be expected that the areas involved in human vocal control have increased access to higher-order auditory information. Following the approach of abstracting to connectivity space (Figure 1), Neubert and colleagues [47^••^] used resting state fMRI in both humans and macaques to investigate whether higher-order auditory cortex (area Tpt) shows preferential connectivity with areas in medial or lateral frontal cortex that they had identified as homologous in the two species. They showed that the balance of auditory connectivity was reversed in the two species, with macaque auditory cortex showing stronger coupling with medial frontal cortex and human auditory cortex showing stronger coupling with ventrolateral frontal cortex. This result could be due to the increased arcuate pathway in humans [48]. More generally, however, it might mean that in humans auditory information is better able to access ventrolateral prefrontal cortical systems for learning arbitrary rules and associations [49,50] which would be a prerequisite for auditory-based language. Consistent with this suggestion, macaques find it much more difficult to learn arbitrary auditory conditional associations than visual conditional associations [51] and have trouble holding auditory information in working memory [52]. In monkeys the connections of auditory cortex with medial frontal cortex might mediate the influence of auditory information on emotional vocalizations and social interaction.

Focusing more explicitly on the laryngeal motor cortex, Kumar and colleagues [53^•^] used tractography to compare the connections of macaque LMC and human dorsal LMC [41]. They compared the connectivity of these areas with known homologs, reporting much stronger connectivity of human LMC with the medial motor network and with inferior parietal areas. This set of results is consistent with models favoring an expanded role of the lateral motor network in humans.

**Figure 3:**
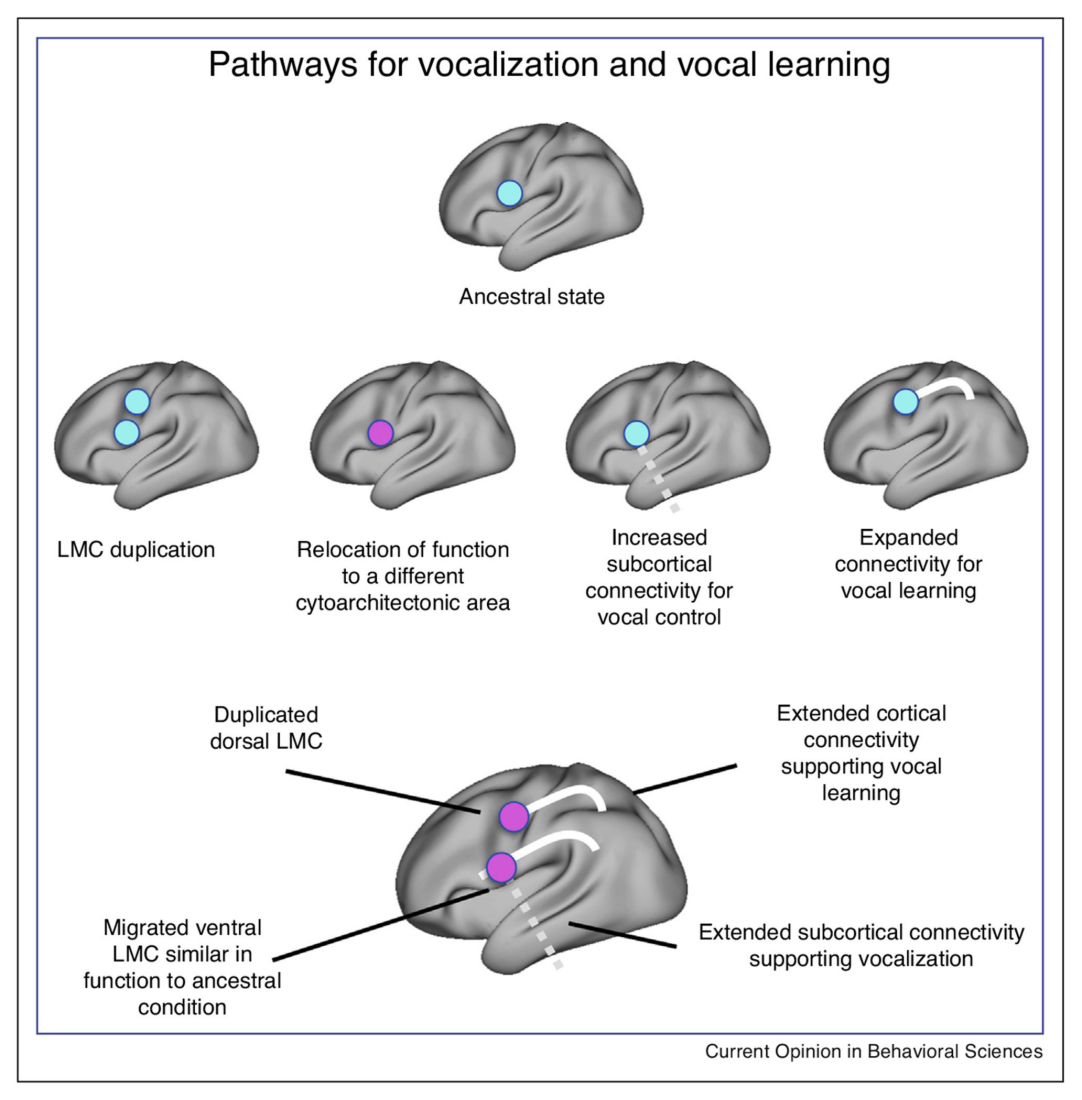
Potential human specializations in pathways for vocalization and vocal learning. Compared to a hypothesized common ancestor of humans and monkeys that has control over laryngeal muscles for functions other than vocalization through a LMC in area 6 (*top*), different research techniques have suggested a number of potential human specializations (*middle*). The presence of multiple larynx representations in the human motor cortex was revealed using stimulation mapping [45], relocation of functional representations of the larynx to cytoarchitectonic area 4 was suggested by functional imaging studies [41], and changes in connectivity consistent with increased vocal control and increased auditory input was shown using diffusion MRI tractography [53^•^]. Although the story is far from complete, viewing these different proposals within a common framework (*bottom*) suggests some hypotheses regarding the nature and function of changes.

The diverse results, often obtained using different techniques, mean that the understanding of the evolution of the human pathways involving LMC is far from complete. However, putting these results within a common framework might help us formulate some hypotheses (Figure 3). The functional characterization of different loci of LMC in the human in a different cytoarchitectonic area than in other primates should be validated by anatomical studies to establish whether these regions indeed are homologs. Second, a complete understanding of the connectivity profile of the two regions will help in understanding their distinct functional contributions. The similarity in function of human and non-human LMC seems greatest for the ventral locus. Its most likely role is increased laryngeal control, although convincing evidence for this in humans is still sparse. Claims of increased connectivity for vocal learning mostly concern the dorsal locus, but again this has yet to be backed up by a complete functional profile. Integration of these different sources of information seems to be a first priority for future empirical studies.

## Conclusions and outlook

Investigating primate cortical specializations is a challenging endeavor. The laborious techniques and the challenges of obtaining data from long living, threatened, and closely related species mean that any comparative dataset inherently presents only a small part of the picture. The pendulum of comparative neuroscience therefore always swings between identifying unique specializations and ignoring differences as the research community attempts to build an understanding of general principles in primate brain organization [54]. However, to understand a behavior as complex as language, both an understanding of shared features — a neurobiological basis for language in the broader sense — and of potential human specializations — a neurobiological basis for language in a narrower sense — are essential.

As we have demonstrated, this endeavor is further complicated by the co-dependencies between changes in cortical organization. An enlarged cortical sheet is likely to lead to increased arealization and a larger brain requires different connectivity to maintain a similar efficiency of inter-regional neural communication [55]. Expansion of the cortex can lead to a change in the location of areas and connections. Therefore, although brain connectivity data have been the most fruitful approach for understanding anatomical specialization for language, these cannot be interpreted without reference to other markers of anatomical organization. We have argued that a full understanding of brain organization is facilitated by placing different types of anatomical data into a common space, allowing one to quantify shared architectural features and deviations of the common plan. This has allowed us to clarify the specializations of the longitudinal connections of the frontal lobe and propose hypotheses for the organization of the cortical control of speech.

Although cross-species studies are still rare, comparative neuroscience using neuroimaging means that data from a much wider range of species than ever before is becoming available to the scientific community [19]. Similarly, techniques for analyzing these data within a common framework are also rapidly developed. This places us in an excellent position to provide novel insights into the evolution of language, an enterprise that has been a major challenge for a wide range of disciplines.
